# Comparison of Ga-68-Labeled Fusarinine C-Based Multivalent RGD Conjugates and [^68^Ga]NODAGA-RGD—*In Vivo* Imaging Studies in Human Xenograft Tumors

**DOI:** 10.1007/s11307-016-0931-3

**Published:** 2016-02-23

**Authors:** Chuangyan Zhai, Gerben M. Franssen, Milos Petrik, Peter Laverman, Dominik Summer, Christine Rangger, Roland Haubner, Hubertus Haas, Clemens Decristoforo

**Affiliations:** 1 Department of Nuclear Medicine, Medical University Innsbruck, Innsbruck, Austria; 2 Department of Nuclear Medicine, Guangdong General Hospital and Guangdong Academy of Medical Sciences, Guangzhou, China; 3 Department of Nuclear Medicine, Radboud University Nijmegen Medical Centre, Nijmegen, The Netherlands; 4 Institute of Molecular and Translational Medicine, Faculty of Medicine and Dentistry, Palacky University, Olomouc, Czech Republic; 5 Division of Molecular Biology, Medical University Innsbruck, Innsbruck, Austria

**Keywords:** Fusarinine C, Integrins, RGD peptides, Gallium-68, Positron emission tomography (PET)

## Abstract

**Purpose:**

Multimeric arginine-glycine-aspartic acid (RGD) peptides have advantages for imaging integrin α_v_β_3_ expression. Here, we compared the *in vitro* and *in vivo* behavior of three different Ga-68-labeled multimeric Fusarinine C-RGD (FSC-RGD) conjugates, whereby RGD was coupled directly, *via* a succinic acid or PEG linker (FSC(RGDfE)_3_, FSC(succ-RGD)_3_, FSC(Mal-RGD)_3_). The positron emission tomography/X-ray computed tomography (PET/CT) imaging properties were further compared using [^68^Ga]FSC(succ-RGD)_3_ with the monomeric [^68^Ga]NODAGA-RGD in a murine tumor model.

**Procedure:**

FSC-RGD conjugates were labeled with Ga-68, and stability properties were studied. For *in vitro* characterization, the partition coefficient, integrin α_v_β_3_ binding affinity, and cell uptake were determined. To characterize the *in vivo* properties, biodistribution studies and microPET/CT were carried out using mice bearing either human M21/M21-L melanoma or human U87MG glioblastoma tumor xenografts.

**Results:**

All FSC-RGD conjugates were quantitatively labeled with Ga-68 within 10 min at RT. The [^68^Ga]FSC-RGD conjugates exhibited high stability and hydrophilic character, with only minor differences between the different conjugates. *In vitro* and *in vivo* studies showed enhanced integrin α_v_β_3_ binding affinity, receptor-selective tumor uptake, and rapid renal excretion resulting in good imaging properties.

**Conclusions:**

The type of linker between FSC and RGD had no pronounced effect on targeting properties of [^68^Ga]FSC-RGD trimers. In particular, [^68^Ga]FSC(succ-RGD)_3_ exhibited improved properties compared to [^68^Ga]NODAGA-RGD, making it an alternative for imaging integrin α_v_β_3_ expression.

**Electronic supplementary material:**

The online version of this article (doi:10.1007/s11307-016-0931-3) contains supplementary material, which is available to authorized users.

## Introduction

Integrins are a family of cation-dependent heterodimeric transmembrane adhesion receptors which are involved in cell-cell, cell-extracellular matrix, and cell-pathogen interactions. They are expressed on a variety of cell types and play an important role in many aspects of tumor development such as tumor-induced angiogenesis, tumor proliferation, and tumor cell migration [1]. Optimized cyclic pentapeptides containing the arginine-glycine-aspartic acid (RGD) motif show high affinity for the integrin α_v_β_3_ as well as remarkable metabolic stability [2, 3]. Versatile RGD-based sequences, acyclic or cyclic, have been labeled with halogens as well as with various radiometals to noninvasively determine the integrin α_v_β_3_ expression [4, 5]. Further studies have demonstrated that multimerization of the RGD motif, i.e., combination of several RGD sequences in one single molecule, can increase binding affinity leading to enhanced target uptake and slower clearance from the targeted tumor [6]. The first successful example allowing noninvasive imaging of integrin α_v_β_3_ expression is [^18^F]Galacto-RGD, which has been tested in preclinical studies and clinical trials with good pharmacokinetics and receptor-specific uptake [7–10]. However, the common problem encountered for F-18 labeling is the complex and time-consuming synthesis of these tracers with moderate yield limiting routine use in the clinical setting.

To achieve an easier and more rapid radiosynthesis, with high specific activity and high yield of the desired radiotracer, an alternative approach is direct labeling with radiometals. Therefore, the RGD peptides were modified with corresponding chelator systems and radiolabeled with appropriate radiometals such as Ga-68, Cu-64, Y-86, and Zr-89 for positron emission tomography (PET) imaging or Tc-99m and In-111 for single-photon emission computed tomography (SPECT) imaging. Of the nuclides mentioned above, Ga-68 has received tremendous attraction as a PET radionuclide due to the ready availability from ^68^Ge/^68^Ga generator systems and its physical characteristics with a half-life of 68 min and a high positron yield of 89 %. This half-life is compatible with the pharmacokinetics of many peptides including RGD peptides. As a result of its significance for PET imaging, several bifunctional chelators have been employed for Ga-68 labeling of biomolecules including the most widely used cyclic 1,4,7,10-tetraazacyclododecane-1,4,7,10-tetraacetic acid (DOTA) derivatives as well as 1,4,7-triaazacyclononane-1,4,7-triacetic acid (NOTA) derivatives (e.g., NODAGA, 1,4,7-triazacyclononane-1,4-bis(acetic acid)-7-(2-glutaric acid)), of which the later are better suited for the small Ga^3+^ ion due to their smaller cavity size. The higher complex stability constant of the NOTA system allows radiolabeling even at room temperature (RT) within minutes. [^68^Ga]NODAGA-RGD is one promising monomeric compound showing excellent *in vitro* and *in vivo* properties [11, 12]. Recently, a multivalent bifuncational chelator named 1,4,7-triazacyclononane-1,4,7-tris[(2-carboxyethyl)methylenephosphinic acid] (TRAP) was developed and Ga-68-labeled TRAP(RGD)_3_ was reported to further improve integrin α_v_β_3_ targeting as compared to [^68^Ga]NODAGA-RGD [13]. Recently, we reported that fusarinine C (FSC), a cyclic hydroxamate siderophore, is a promising Ga-68 and Zr-89 binding bifunctional chelator [14–17]. FSC possesses three primary amine functions allowing conjugation of up to three targeting vectors, thereby facilitating the application of the multimerization approach. FSC-based multimeric RGD conjugates (FSC(succ-RGD)_3_, FSC(RGDfE)_3_, and FSC(Mal-RGD)_3_) were previously synthesized *via* different conjugation strategies [14, 16]. *In vitro* and *in vivo* evaluation of Zr-89-labeled FSC-RGD conjugates demonstrated the superiority of FSC as a bifunctional chelator for Zr-89 as well as the receptor specificity of [^89^Zr]FSC-RGD conjugates [16]. However, the long physical half-life of Zr-89 (78.4 h) may be considered nonideal for the biological half-lives of small RGD peptides which range from several minutes to hours. By contrast, the 68-min half-life of Ga-68 matches well with the biological half-lives of the cyclic RGD peptides. As an excellent Ga-68 chelator, FSC complexation of Ga-68 occurs at RT resulting in high RCP and specific activity within minutes [15].

In this study, FSC(succ-RGD)_3_, FSC(RGDfE)_3_, and FSC(Mal-RGD)_3_ were labeled with Ga-68 and the comparison of *in vitro* and *in vivo* properties including PET/CT imaging of these compounds in a human melanoma tumor model is reported for the first time. PET/CT imaging properties of [^68^Ga]FSC(succ-RGD)_3_ were further compared with the monomeric [^68^Ga]NODAGA-RGD in an alternative human glioblastoma tumor model.

## Materials and Methods

### General

All described substances and solvents were of reagent grade and were used without further purification. FSC(succ-RGD)_3_, FSC(RGDfE)_3_, and FSC(Mal-RGD)_3_ were synthesized as described earlier [16]. Milli-Q water was used for preparing all reagent solutions. Human melanoma M21 and M21-L cells were a kind gift from D. A. Cheresh (Department of Pathology and the Moores Cancer Center, University of California San Diego, La Jolla, CA, USA), and human glioblastoma U87MG cells were obtained from American Type Culture Collection (ATCC, Manassas, VA, USA). Elution of the Ga-68 generator (IGG100, Eckert & Ziegler Strahlen- und Medizintechnik AG, Berlin, Germany, nominal activity 1850 MBq) was performed with 0.1-M HCl solution (Rotem Industries Ltd., Beer-Sheva, Israel).

### High-Performance Liquid Chromatography

An UltiMate 3000 (Thermo Fisher Scientific, Vienna, Austria) high-performance liquid chromatography (HPLC) system consisting of an UltiMate 3000 RS pump, a column oven (temperature setting 25 °C) UV-vis variable wavelength detector (220 nm), and a radioactivity detector (Raytest GABI, Raytest, Straubenhardt, Germany) was used; gradient: acetonitrile (CH_3_CN)/H_2_O/0.1 % trifluoroacetic acid (TFA) gradient: 0–0.5 min 0 % CH_3_CN, 0.5–7.0 min 0–55 % CH_3_CN

### Ga-68 Radiolabeling

The fraction containing the highest Ga-68 activity (approximately 400–500 MBq in 0.1 M HCl) was collected, and 110 μl 1.9-M NaOAc solution was added to adjust pH to 4.5. After briefly shaking, 100 μl of the corresponding solution was mixed with 30 μl of the FSC-RGD conjugates (1 μg/μl in water; FSC(succ-RGD)_3_, 10.8 nmol; FSC(RGDfE)_3_, 12.0 nmol; or FSC(Mal-RGD)_3_, 7.9 nmol) and incubated at RT for 10 min. Analytical HPLC was used to confirm quantitative labeling. Subsequently, the labeling solution was diluted with phosphate‐buffered saline (PBS) and used for *in vitro* and *in vivo* studies without further purification.

The labeling of NODAGA-RGD with Ga-68 was carried out as described previously [11]. Briefly, to 100 μl of Ga-68 eluate (approximately 40–50 MBq in 0.1 M HCl), 10 μl of NODAGA-RGD (1 μg/μl in water, 10.4 nmol) was added and the pH of the reaction solution was adjusted to 5 using 1.9-M NaOAc solution. The solution was allowed to react for 15 min at RT. Subsequently, the labeling solution with a radiochemical yield (RCY) higher than 96 % was diluted with PBS and used without further purification.

### Stability Studies

The stability of [^68^Ga]FSC(succ-RGD)_3_, [^68^Ga]FSC(RGDfE)_3_, and [^68^Ga]FSC(Mal-RGD)_3_ was evaluated by incubating the radiotracers in PBS, EDTA solution (pH 7; >1000-fold molar excess of EDTA), and fresh human serum for up to 120 min, respectively. At selected time points, aliquots of PBS or EDTA solution were analyzed directly *via* RP-HPLC, while serum aliquots were mixed with equal volumes of CH_3_CN, vortexed, and centrifuged at 20,000 relative centrifugal force (rcf) for 2 min. Before analysis, the precipitate was washed three times using CH_3_CN.

### Distribution Coefficient (logD)

A volume of 500 μl PBS including 0.5 MBq radioligand (approximately 0.5 μg peptide) was combined with 500 μl octanol; the mixture was vortexed for 15 min and centrifuged for 2 min at 2000 rcf. From each phase, an aliquot (50 μl) was pipetted out and measured in a 2480 Wizard2 Automatic Gamma Counter (PerkinElmer, Vienna, Austria). Each measurement was repeated five times. LogD value was calculated as the average log ratio of the radioactivity in the organic fraction and the PBS fraction.

### Binding Affinity for Immobilized Integrin α_v_β_3_ (Half Maximal Inhibitory Concentration Value)

*In vitro* binding affinities of cyclo(-Arg-Gly-Asp-DTyr-Val-) (c(RGDyV)) and the three FSC-RGD conjugates were determined by using isolated integrin α_v_β_3_ (Millipore-Chemicon, Temecula, CA, USA) and I-125-labeled c(RGDyV) as radioligand. ([^125^I]c(RGDyV) was produced as described in [18]). To integrin α_v_β_3_, coated overnight at 4 °C onto 96-well plates (Nunc, Thermo Fisher Scientific, Vienna, Austria), [^125^I]c(RGDyV) and the corresponding peptides in increasing concentrations from 0.001 to 100 nM were added. Plates were washed, and integrin bound activity was recovered with 2-M NaOH solution and counted in a gamma counter. OriginPro 8.5 software (Northampton, MA, USA) was used to calculate half maximal inhibitory concentration (IC_50_) values in three independent measurements.

### Cell Culture

All cell lines were grown to confluence at 37 °C in a humidified atmosphere of 95 % air/5 % carbon dioxide and split approximately every 48 h. U-87 MG cells were cultured in tissue culture flasks (Cellstar^®^; Greiner Bio-One, Kremsmuenster, Austria) using EMEM supplemented with 10 % volume/volume (*v*/*v*) heat-inactivated fetal bovine serum (FBS), 1 % *v*/*v* penicillin/streptomycin/glutamine solution (PSG), 1 % *v*/*v* sodium pyruvate solution, and 1 % *v*/*v* nonessential amino acid solution. M21 cells were grown using DMEM supplemented with 10 % *v*/*v* FBS and 1 % *v*/*v* PSG solution. M21-L cells were maintained in germ count dishes (Greiner Bio-One) in RPMI 1640 cell culture medium containing 10 % FBS and 1 % PSG.

### Internalization Assay

M21 cells (integrin α_v_β_3_ positive) were diluted with RPMI 1640 (Gibco, Invitrogen Corporation, Paisley, UK) containing 1 % glutamine (*m*/*v*), 1 % bovine serum albumin (BSA) (*m*/*v*), CaCl_2_ (1 mM), MgCl_2_ (1 mM), and MnCl_2_ (10 mM) to a concentration of 2.0 × 10^6^ cells/ml. One-milliliter aliquots in Eppendorf tubes were incubated at 37 °C for 1 h in triplicates, and [^68^Ga]FSC(succ-RGD)_3_, [^68^Ga]FSC(RGDfE)_3_, or [^68^Ga]FSC(Mal-RGD)_3_ (approximately 1.5 × 10^6^ cpm, 1.2 μg peptides) in PBS with 0.5 % BSA (150 μl, total series) or with 10 μM c(RGDyV) in PBS/0.5 % BSA (150 μl, nonspecific series) at 37 °C was added. After 90-min incubation was stopped by centrifugation, the medium was removed followed by washing (twice) with ice-cold Tris-buffered saline. After incubation in acid wash buffer (20 mM acetate buffer, pH 4.5) at 37 °C for 5 min (two times) and centrifugation, membrane bound activity was collected in plastic vials. Internalized radioligand fraction was collected after lysing cells by addition of 2 M NaOH. The internalized activity was expressed as percentage of total activity per milligram protein after spectrophotometric determination of protein (Bradford assay) in the NaOH fraction.

### *Ex Vivo* Biodistribution Studies

All animal experiments were approved by the Austrian Ministry of Science (BMWF-66.011/000604-II/3b/2012 and BMWFW-66.011/0049-WF/II/3b/2014) and the Czech Ministry of Education Youth and Sports (MSMT-22421/2013-12) as well as by the institutional Animal Welfare Committee of the Faculty of Medicine and Dentistry of Palacky University in Olomouc and conducted in compliance with the Austrian and Czech animal protection laws.

For the induction of tumor xenografts, female, athymic BALB/c nude mice (Charles River Laboratories) were injected subcutaneously with either 5 × 10^6^ integrin α_v_β_3_-positive M21 cells into the right hind limb and with 5 × 10^6^ integrin α_v_β_3_-negative M21-L cells (negative control) into the left hind limb of the same mouse. Approximately 3 weeks after inoculation, the tumors had reached a volume of 0.3–0.6 cm^3^. On the day of the experiment, [^68^Ga]FSC(succ-RGD)_3_, [^68^Ga]FSC(RGDfE)_3_, or [^68^Ga]FSC(Mal-RGD)_3_ (∼0.5 MBq/mouse, ∼0.5 μg peptide, respectively) was intravenously injected in the lateral tail vein. Mice were euthanized by cervical dislocation at 1 and 2 h (only for [^68^Ga]FSC(succ-RGD)_3_) after injection. Organs (spleen, pancreas, stomach, intestine, kidney, liver, heart, and lung), blood, muscle tissue, and tumors were dissected and weighed. Activity of the different samples was measured in the gamma counter. Results were expressed as percentage of injected dose per gram tissue (% ID/g).

### MicroPET/CT Imaging

To compare the imaging properties of [^68^Ga]FSC(succ-RGD)_3_, [^68^Ga]FSC(RGDfE)_3_, and [^68^Ga]FSC(Mal-RGD)_3_, microPET/CT imaging experiments were conducted on an Inveon microPET/CT scanner (Siemens Preclinical Solutions, Knoxville, USA). BALB/c nude mice bearing M21-positive tumor xenografts were administered with [^68^Ga]FSC(succ-RGD)_3_ (or [^68^Ga]FSC(RGDfE)_3_, [^68^Ga]FSC(Mal-RGD)_3_, ∼5 MBq/mouse, ∼5 μg peptide) *via* intravenous (i.v.) injection. The body temperature of mice was maintained at 37 °C on the scanner bed and in the incubation chambers using heating pads. Dynamic 10-min duration PET scans were performed for 50 min while static PET scans (10 min) were performed at 1-h post-injection (p.i.), followed by a 25-min CT scan (spatial resolution, 113 μm; 80 kV, 500 mA). The mice remained under general anesthesia (isoflurane/O_2_) during the time between injection and imaging. The microPET/CT scans were reconstructed with Inveon Acquisition Workplace software (version 1.5; Siemens Preclinical Solutions), using a three-dimensional fast maximum a posteriori algorithm with the following parameters: matrix 256 × 256 × 161; pixel size 0.4 × 0.4 × 0.8 mm, and *β*-value of 1.5-mm resolution with uniform variance. Spherical isocontours were set at 50 % of the maximal pixel value to measure uptake values (Bq/ml) in kidneys, heart, and tumor.

To investigate the capabilities of binding to the integrin α_v_β_3_ receptors in the different tumor xenograft models and, at the same time, compare the imaging properties of [^68^Ga]FSC(succ-RGD)_3_ and [^68^Ga]NODAGA-RGD, two U87MG tumor-bearing mice were administered retro-orbitally (r.o.) [19] under general anesthesia with [^68^Ga]FSC(succ-RGD)_3_ and [^68^Ga]NODAGA-RGD each other (∼6 MBq/mouse, ∼5 μg peptide) at continued days. For the induction of tumor xenografts, female, athymic BALB/c nude mice (AnLab, Prague, Czech Republic) were injected subcutaneously with 5 × 10^6^ U87MG cells into the right flank. The tumors were allowed to grow until they had reached a volume of 0.5 to 0.8 cm^3^. Imaging experiments were conducted on an Albira microPET/SPECT/CT imaging system (Bruker Biospin Corporation, Woodbridge, CT, USA). MicroPET/CT images were acquired under general anesthesia (isoflurane/O_2_) and heating at 37 °C. PET data for each mouse was recorded *via* either dynamic imaging for 90 min (5-min PET scan per frame) or static scans (a 5-min PET scan (axial FOV 148 mm) was performed, followed by a 25-min CT scan (axial FOV 65 mm, 45 kVp, 400 μA, at 600 projections)) at 90 min p.i. The microPET/CT scans were reconstructed with Albira software (Bruker Biospin Corporation, Woodbridge, CT, USA) using maximum likelihood expectation maximization (MLEM) and filtered backprojection (FBP) algorithms. Dead time, decay correction, attenuation correction, and normalization were applied to all PET data. The uptake values (%ID/g) in kidneys, heart, and tumor were analyzed by drawing standardized volumes of interest.

### Statistical Analysis

Statistical analysis was performed using SPSS 17.0 software. The biodistribution data was analyzed using Student’s *t* test. The level of significance was set at *P* < 0.05.

## Results

### Radiolabeling and Stability Studies

All FSC-RGD conjugates were labeled with Ga-68 using NaOAc buffer at pH 4.5 at RT within 10 min resulting in almost quantitative labeling efficiency (>98 %) and specific activities ranging from 2.5 to 4 GBq/μmol. For all experiments, the labeling solution was used without further purification. Stability studies revealed high stability for all three compounds in all examined media for up to 2 h, indicating that the different conjugation strategies had no detectable influence on the stability of Ga-68-labeled FSC conjugates.

### *In Vitro* Characterization

All three Ga-68-labeled FSC-RGD conjugates showed hydrophilic properties following the order of [^68^Ga]FSC(RGDfE)_3_ (−3.8 ± 0.2) > [^68^Ga]FSC(succ-RGD)_3_ (−3.6 ± 0.1) > [^68^Ga]FSC(Mal-RGD)_3_ (−3.4 ± 0.0).

Binding of [^125^I]c(RGDyV to integrin α_v_β_3_ was successfully displaced by all FSC-RGD conjugates and c(RGDyV) in a concentration-dependent manner. IC_50_ values were 1.8 ± 0.7 nM for FSC(succ-RGD)_3_, 2.1 ± 0.9 nM for FSC(RGDfE)_3_, and 3.4 ± 0.6 nM for FSC(Mal-RGD)_3_, indicating enhanced binding affinities as compared to c(RGDyV) (6.2 ± 1.6 nM).

**Fig. 1:**
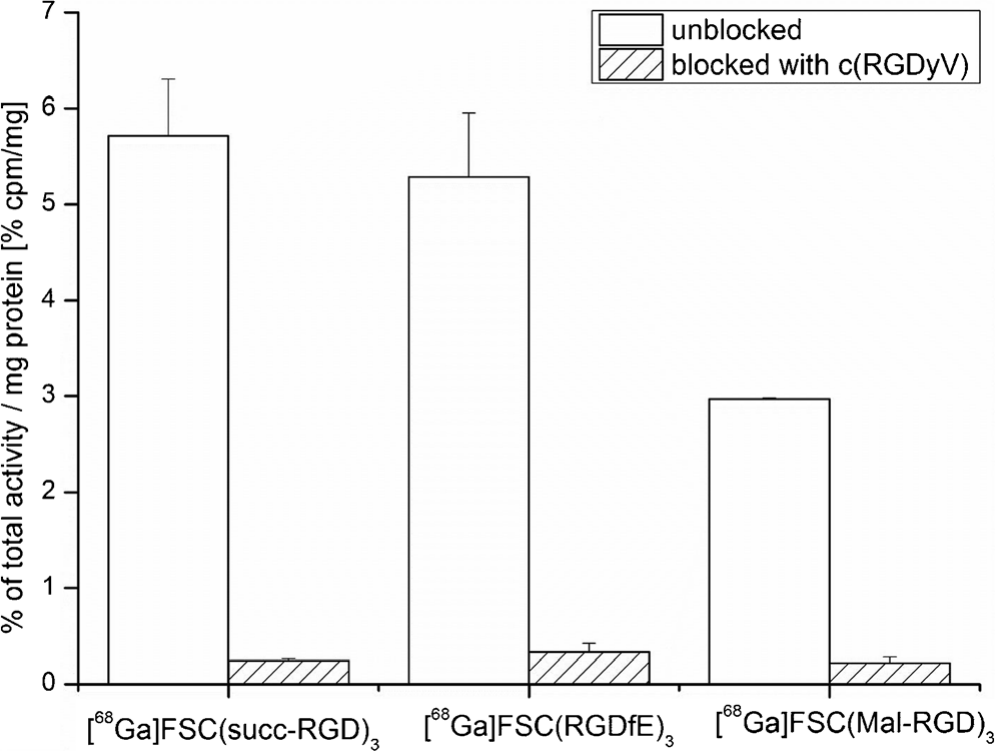
Internalization of [^68^Ga]FSC(succ-RGD)_3_, [^68^Ga]FSC(RGDfE)_3_, and [^68^Ga]FSC(Mal-RGD)_3_ in integrin α_v_β_3_-positive M21 tumor cells.

The internalization ability of Ga-68-labeled FSC-RGD conjugates is shown in Fig. 1. The internalized activities were 5.7 ± 0.6 % for [^68^Ga]FSC(succ-RGD)_3_, 5.3 ± 0.7 % for [^68^Ga]FSC(RGDfE)_3_, and 3.0 ± 0.0 % for [^68^Ga]FSC(Mal-RGD)_3_ of total activity per milligram protein (% cpm/mg) after 90-min incubation. The corresponding activities were reduced to 0.2 to 0.4 % cpm/mg of the reference activity *via* addition of excess of c(RGDyV) which demonstrated receptor-specific internalization.

### *Ex Vivo* Biodistribution Studies

**Fig. 2:**
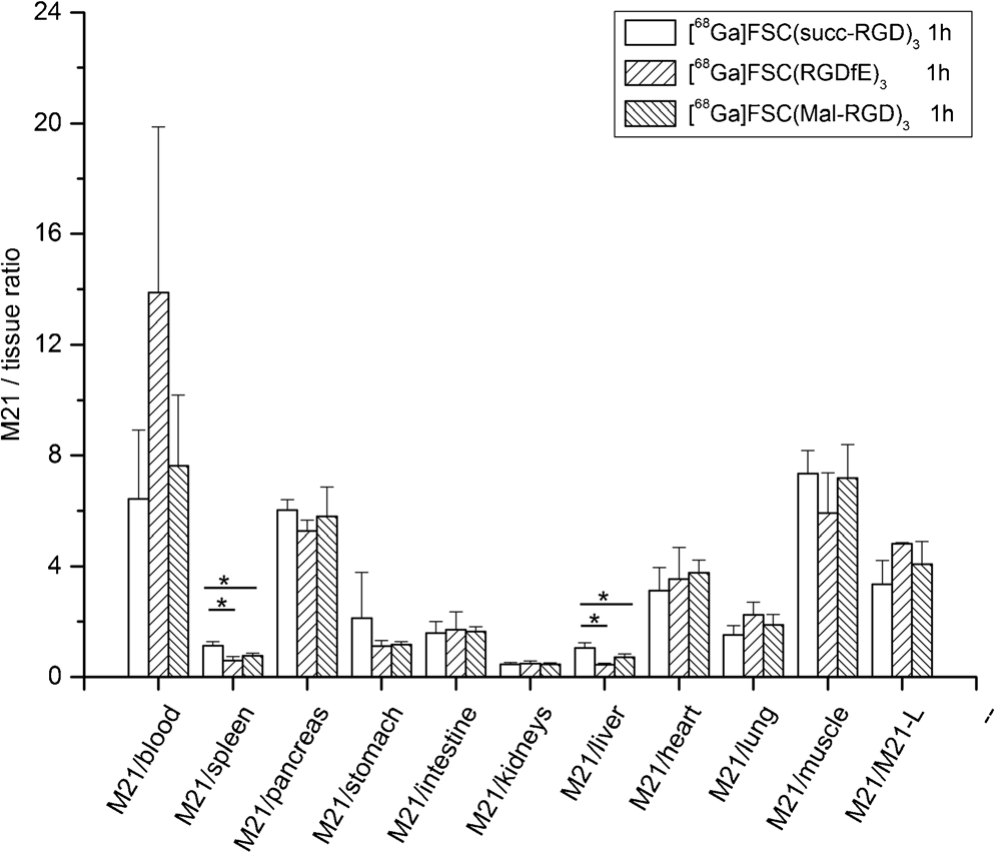
Comparison of M21 melanoma to tissue ratios of [^68^Ga]FSC(succ-RGD)_3_, [^68^Ga]FSC(RGDfE)_3_, and [^68^Ga]FSC(Mal-RGD)_3_ at 1 h p.i. M21/M21-L human melanoma xenografted nude mice were used. Significant differences in uptake are marked with an *asterisk* (*P* < 0.05).

The data for the biodistribution studies of all three Ga-68-labeled FSC-RGD conjugates in BALB/c nude mice bearing both integrin α_v_β_3_-positive (M21) as well as integrin α_v_β_3_-negative (M21-L) human melanoma tumors 1 h p.i. are presented in Table 1 and the tumor to tissue ratios in Fig. 2. The activity accumulation in the M21 tumor for the three compounds is decreasing in the order: [^68^Ga]FSC(RGDfE)_3_ (5.49 ± 0.94 %ID/g) > [^68^Ga]FSC(succ-RGD)_3_ (3.69 ± 0.94 %ID/g) > [^68^Ga]FSC(Mal-RGD)_3_ (2.62 ± 0.27 %ID/g). The receptor-specific uptake is demonstrated by the lower uptake found in the receptor-negative M21-L tumor (1.10 ± 0.30, 1.25 ± 0.06, 0.68 ± 0.09 %ID/g, respectively) resulting in comparable M21/M21-L ratios for all three compounds.

**Table 1:** Biodistribution of [^68^Ga]FSC(succ-RGD)_3_, [^68^Ga]FSC(RGDfE)_3_, and [^68^Ga]FSC(Mal-RGD)_3_ at 1 h p.i. in BALB/c nude mice bearing integrin α_v_β_3_-positive tumors (M-21) on the right flank and integrin α_v_β_3_-negative tumors (M21-L) on the left flank

Organ/tissue	[^68^Ga]FSC(succ-RGD)_3_ (% ID/g)	[^68^Ga]FSC(RGDfE)_3_ (% ID/g)	[^68^Ga]FSC(Mal-RGD)_3_ (% ID/g)
Blood	0.63 ± 0.26	0.40 ± 0.08	0.36 ± 0.08
Spleen	3.27 ± 0.64	9.13 ± 0.27	3.48 ± 0.56
Pancreas	0.61 ± 0.13	1.04 ± 0.12	0.46 ± 0.07
Stomach	2.02 ± 1.35	4.76 ± 0.25	2.24 ± 0.17
Intestine	2.41 ± 0.80	3.21 ± 0.48	1.61 ± 0.30
Kidneys	8.12 ± 1.09	11.52 ± 1.87	5.77 ± 0.89
Liver	3.54 ± 0.63	12.09 ± 1.34	3.77 ± 0.95
Heart	1.21 ± 0.28	1.52 ± 0.13	0.70 ± 0.05
Lung	2.42 ± 0.22	2.48 ± 1.07	1.42 ± 0.26
Muscle	0.52 ± 0.19	0.94 ± 0.09	0.37 ± 0.08
M21	3.69 ± 0.94	5.49 ± 0.94	2.62 ± 0.27
M21-L	1.10 ± 0.30	1.25 ± 0.06	0.68 ± 0.09

Each data point represents an average of three to four animals

A statistical significant difference in the biodistribution data of the three radioligands was observed only for spleen and liver. [^68^Ga]FSC(succ-RGD)_3_ showed superior tumor to tissue ratios in aforementioned organs compared to the other two radioligands.

### MicroPET/CT Studies

**Fig. 3:**
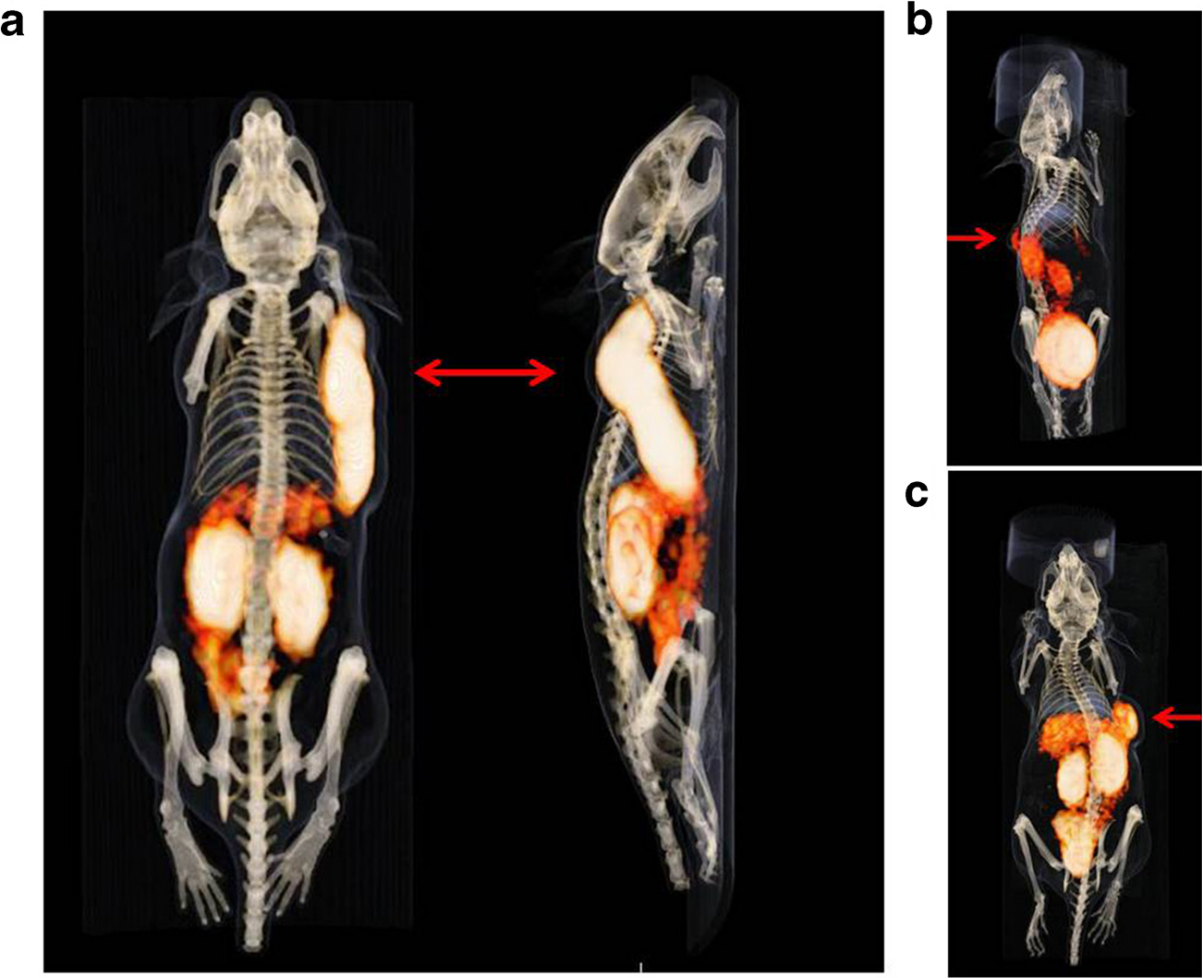
Three-dimensional volume projections of fused microPET/CT static images of a nude mouse bearing a M21 xenograft tumor at 1 h p.i. **a** [^68^Ga]FSC(succ-RGD)_3_, **b** [^68^Ga]FSC(RGDfE)_3_, and **c** [^68^Ga]FSC(Mal-RGD)_3_. *Red arrow*: integrin α_v_β_3_-positive M21 tumor. Injected dose per mouse 5 MBq.

**Fig. 4:**
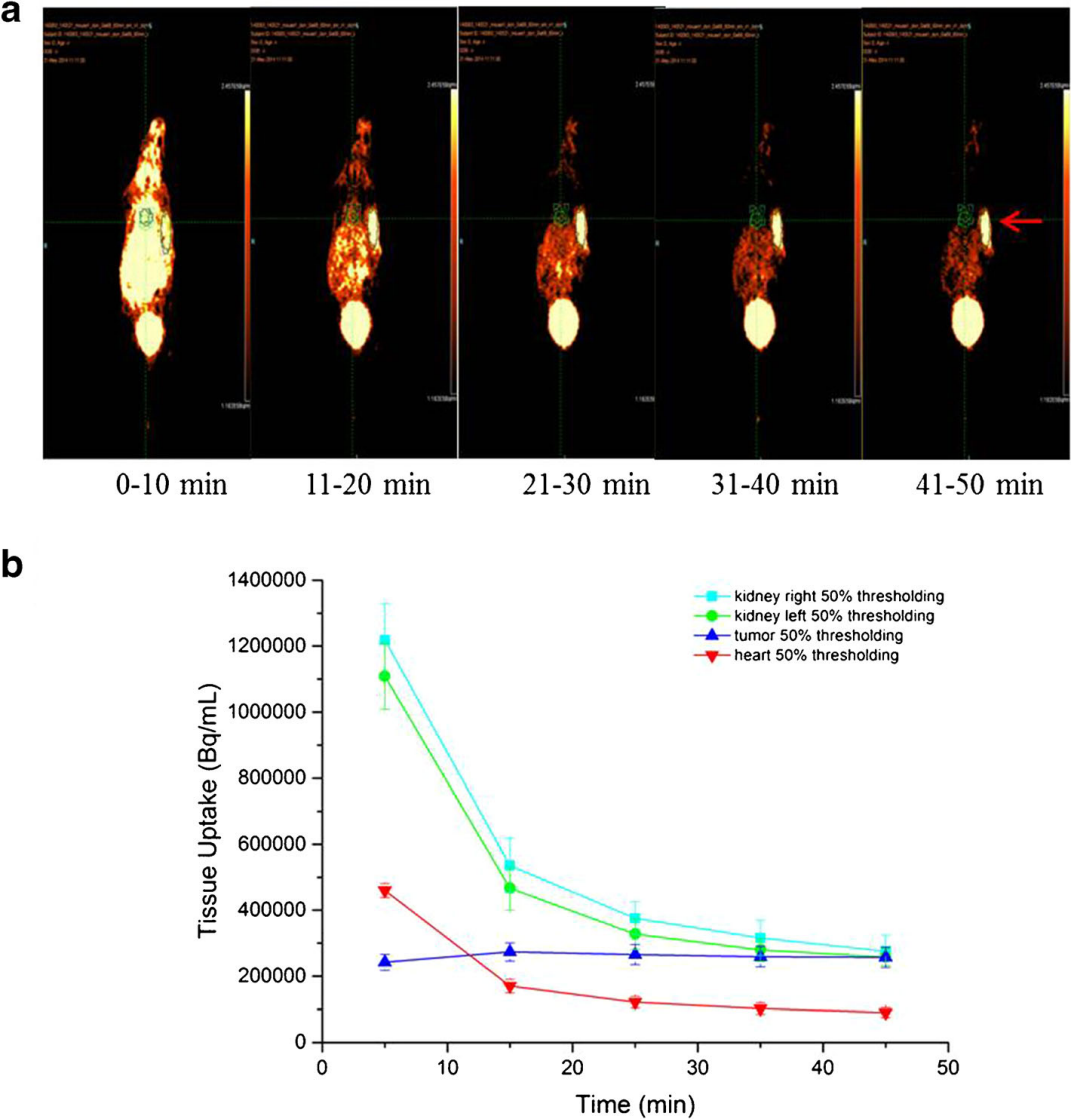
**a** Dynamic PET imaging of [^68^Ga]FSC(succ-RGD)_3_ in a nude mouse bearing a M21 tumor xenograft. Injected dose 5 MBq. *Red arrow*: integrin α_v_β_3_-positive M21 tumor. **b** The dynamic clearance curves of [^68^Ga]FSC(succ-RGD)_3_ in selective tissues over 45 min.

**Fig. 5:**
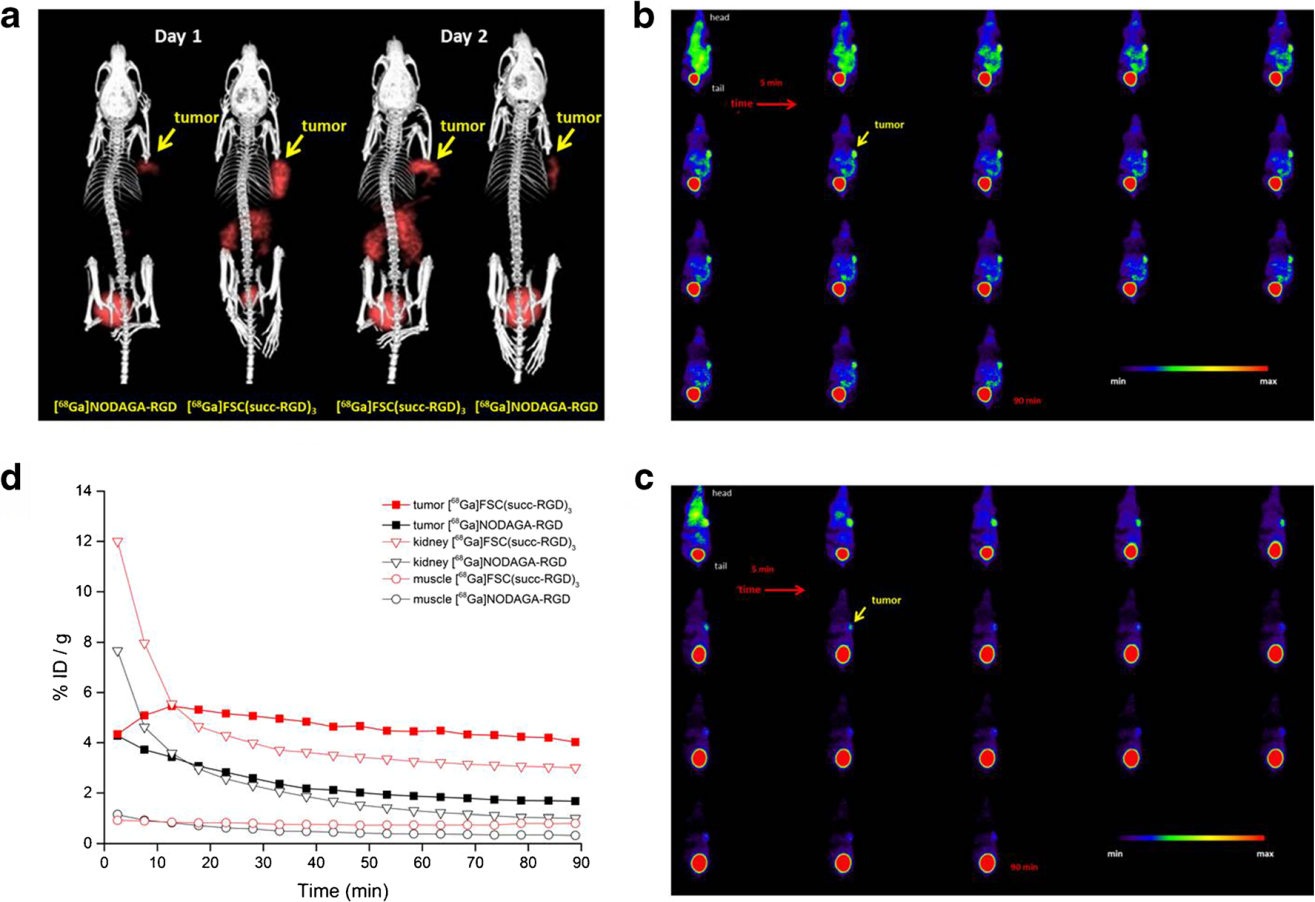
**a** Three-dimensional volume rendered projections of fused microPET/CT static images for [^68^Ga]FSC(succ-RGD)_3_ and [^68^Ga]NODAGA-RGD at 90 min p.i. (two mice imaged per day on two consecutive days where on day 1, mouse 1 received tracer A and mouse 2 tracer B and vice versa)). **b** Dynamic PET images of [^68^Ga]FSC(succ-RGD)_3_ and **c** [^68^Ga]NODAGA-RGD in the same mouse on two consecutive days. Scan duration 5-min PET scan per frame (18 frames). **d** Quantitative ROI analysis of uptake of [^68^Ga]FSC(succ-RGD)_3_ and [^68^Ga]NODAGA-RGD in tumor, kidneys, and muscle of the same BALB/c mice for 90 min p.i. on two consecutive days. Mice bearing U87MG xenograft tumors; injected dose 6 MBq.

MicroPET/CT studies with BALB/c nude mice bearing α_v_β_3_-positive tumors were performed in order to compare the imaging properties and the *in vivo* pharmacokinetics of [^68^Ga]FSC(succ-RGD)_3_, [^68^Ga]FSC(RGDfE)_3_, and [^68^Ga]FSC(Mal-RGD)_3_. All three radioligands showed receptor-specific uptake accompanied by predominantly renal elimination at 1 h p.i., resulting in good image contrasts (Fig. 3). In the static image of [^68^Ga]FSC(succ-RGD)_3_, the α_v_β_3_-positive tumor is clearly visualized with high resolution whereas unspecific uptake of the radioligand is minimal with the exception of the kidneys. To further evaluate the imaging properties of [^68^Ga]FSC(succ-RGD)_3_, dynamic PET imaging was performed and the time-activity curves of tumor, kidney, and heart were extracted (Fig. 4). The dynamic images allow visualization of the receptor-positive tumor as early as 10 min p.i., confirming the rapid accumulation of [^68^Ga]FSC(succ-RGD)_3_ in the tumor. The time-activity curves of the kidneys and heart from dynamic PET data showed rapid clearance of [^68^Ga]FSC(succ-RGD)_3_ from the circulation while the time-activity curve of the tumor showed a clear tracer retention. To directly compare the imaging properties of [^68^Ga]FSC(succ-RGD)_3_ and [^68^Ga]NODAGA-RGD radiotracers, static (Fig. 5a) and dynamic PET (Fig. 5b, c) imaging using mice bearing U87MG xenograft tumors was carried out with both radiotracers. Three-dimensional volume projections of fused microPET/CT static images for [^68^Ga]FSC(succ-RGD)_3_ and [^68^Ga]NODAGA-RGD were carried out at 90 min p.i. by two mice imaged per day on two consecutive days where on day 1, mouse 1 received tracer A and mouse 2 tracer B and vice versa. The dynamic microPET studies were carried out in the same mouse by 5 min PET scan per frame over 90 min p.i. Both static and dynamic PET images showed uptake in U87MG tumors of both radiotracers, however, with improved tumor delineation for [^68^Ga]FSC(succ-RGD)_3_. From the quantitative region of interest (ROI) analysis, uptake values of [^68^Ga]FSC(succ-RGD)_3_ in tumor, kidneys, and muscle were 3.8, 3.0, and 0.78 %ID/g at 90 min p.i., while the corresponding data for [^68^Ga]NODAGA-RGD was 1.6, 1.0, and 0.32 %ID/g, respectively. Despite an approximately 3-fold higher tumor uptake, tumor to muscle (4.9 *vs*. 5) and tumor to kidney ratios (1.3 *vs*. 1.6) are comparable.

## Discussion

Many radiolabeled cyclic RGD peptides have been evaluated as SPECT and PET radiotracers for imaging integrin α_v_β_3_ expression. Among these tracers, radiolabeled multivalent RGD peptides, mainly dimeric and tetrameric derivatives, have been studied extensively on the basis of the multivalency concept [6, 20]. These multimeric RGD peptides exhibited improved receptor affinity due to the avidity effect or simultaneous multivalent binding and prolonged target retention due to increased tracer concentration compared to that of their monomeric analogues. However, comparison of dimeric and tetrameric cyclic RGD peptides showed superiority of dimers over tetramers with respect to tumor to tissue ratios [20]. The longer retention in nontarget tissues, mainly due to higher molecular weight, results in slower elimination which could not be compensated for by the higher tumor uptake of tetrameric constructs. Recently, TRAP(RGD)_3_ was reported as a trimeric RGD peptide for ^68^Ga labeling and showed comparable tissue uptake to monomeric analogues but significant enhanced tumor uptake providing the potential for imaging of low-level integrin expression in tissues [13]. Based on these findings, great attention should be taken to reach a subtle balance between the tumor uptake and tumor to tissue ratios.

Recently, we reported three FSC-based trimeric RGD conjugates [16] labeled with Zr-89 showing excellent *in vitro* stability. PET imaging of [^89^Zr]FSC(succ-RGD)_3_ in BALB/c nude mice demonstrated receptor specificity of FSC-RGD conjugates with high accumulation in α_v_β_3_-positive tumors whereas α_v_β_3_-negative tumors were not visualized during the 24 h monitoring period. To further investigate these promising properties of these FSC-RGD conjugates, we now describe the characterization and, in particular, studies on the imaging properties of Ga-68-labeled FSC-RGD conjugates in two tumor models including static and dynamic imaging.

In the present study, all three FSC-RGD conjugates, namely FSC(succ-RGD)_3_, FSC(RGDfE)_3_, and FSC(Mal-RGD)_3_, were successfully labeled with Ga-68 following the protocol that we reported previously [15]. Quantitative labeling of FSC-RGD conjugates was performed at pH 4.5 at RT within 10 min. The logD values of three Ga-68-labeled FSC conjugates ranged from −3.4 to −3.8, displaying a more hydrophilic character compared to their Zr-89-labeled counterparts which ranged from −2.9 to −3.0 [16]. The difference of lipophilicity may result from the different net charges of the radiolabeled compounds (the net charge of [^68^Ga^3+^]FSC chelate itself is 0 while that of [^89^Zr^4+^]FSC chelate is +1).

Competition studies demonstrated the enhanced binding affinity of the multimeric FSC-RGD conjugates compared with the monomeric c(RGDyV), with highest affinities for FSC(succ-RGD)_3_ and FSC(RGDfE)_3_. This was further confirmed by internalization assays with the Ga-68-labeled FSC-RGD conjugates which result in much higher internalization compared to [^68^Ga]NODAGA-RGD. The differences in binding affinity and internalization properties between the different conjugates can be attributed to the influence of the different linkers (e.g., length and hydrophilicity), whereby FSC(Mal-RGD)_3_ with the longest linker showed the lowest affinity and lowest internalization values, whereas FSC(succ-RGD)_3_ and FSC(RGDfE)_3_ were comparable. Compared with Zr-89-labeled FSC-RGD conjugates [16], the same trend of internalization is confirmed even though the Zr-89-labeled compounds showed slightly lower internalization values than their Ga-68 counterparts.

The biodistribution data of [^68^Ga]FSC(succ-RGD)_3_, [^68^Ga]FSC(RGDfE)_3_, and [^68^Ga]FSC(Mal-RGD)_3_ in BALB/c nude mice bearing integrin α_v_β_3_-positive M21 tumors at 1 h p.i. were directly compared. All three compounds exhibited target-specific binding with enhanced tumor uptakes compared to [^68^Ga]NODAGA-RGD (1.3 %ID/g [14]) and relative low uptakes in nontarget tissue. PET studies revealed high-contrast images of the α_v_β_3_-positive M21 tumor at 1 h, confirming the biodistribution data (Table 1 and Fig. 3). Among the three compounds, [^68^Ga]FSC(succ-RGD)_3_ showed superior tumor to tissue ratios for spleen and liver, which is the reason that [^68^Ga]FSC(succ-RGD)_3_ was chosen for further studies. Comparing the tumor to tissue ratios of [^68^Ga]FSC(succ-RGD)_3_ at 1 and 2 h p.i. (Fig. S1), increased tumor to background ratios were found for the later time point providing the potential for delayed imaging.

Biodistribution and tumor targeting of [^68^Ga]FSC(succ-RGD)_3_ were very comparable to its Zr-89 counterpart [16]. Tumor and organ uptake values at 1 h p.i. were not significantly different, an exception was a significantly higher activity of the [^68^Ga]FSC-RGD conjugate in blood, liver, spleen, and α_v_β_3_-negative M21-L tumor, the later tissues, however, minor in terms of absolute values. This could be explained by a different biological behavior and binding of released Ga-68 by transferrin resulting in longer blood circulation. On the other hand, no indication of differences in *in vitro* stability or protein binding of the complexes was found and image contrast in microPET images was excellent. Therefore, these differences could also be related to the different net charges of the FSC chelate.

In Table 2, tumor to tissue ratios of [^68^Ga]FSC(succ-RGD)_3_ are compared with the literature data of [^68^Ga]TRAP(RGD)_3_ and [^68^Ga]NODAGA-RGD. Overall, very similar ratios are observed for [^68^Ga]FSC(succ-RGD)_3_ and [^68^Ga]TRAP(RGD)_3_ except for the tumor to blood ratio. [^68^Ga]TRAP(RGD)_3_ exhibited higher ratios than [^68^Ga]FSC(succ-RGD)_3_ at 1 and 2 h p.i., which is probably due to a faster clearance from blood of [^68^Ga]TRAP(RGD)_3_. Notably, the tumor to kidney ratios of [^68^Ga]FSC(succ-RGD)_3_ and [^68^Ga]TRAP(RGD)_3_ are almost the same, indicating that the relative high kidney excretion is mainly determined by the multimeric RGD peptide part and not the chelator moiety. However, whereas for [^68^Ga]TRAP(RGD)_3_, tumor to background ratios remained constant over time, for our compound, tumor to background ratios improved up to 2 h p.i. again indicating an advantage of PET imaging at late time points for [^68^Ga]FSC(succ-RGD)_3_. In particular, much higher tumor to muscle ratios were observed (14.4 *vs*. 6.9 for [^68^Ga]TRAP(RGD)_3_). Compared with the data found for [^68^Ga]NODAGA-RGD, [^68^Ga]FSC(succ-RGD)_3_ exhibited different tumor to tissue ratios for blood and kidney. Even though not statistically significant, [^68^Ga]NODAGA-RGD exhibited 1.8-fold higher tumor to blood and 2-fold higher tumor to kidney ratios 1 h p.i. These differences could be attributed to a slower blood clearance rate and kidney excretion of [^68^Ga]FSC(succ-RGD)_3_. However, they were not confirmed in PET imaging, where comparable tumor to tissue ratios were found. The direct comparison of [^68^Ga]FSC(succ-RGD)_3_ with [^68^Ga]NODAGA-RGD in mice bearing U87MG xenograft tumors by static and dynamic PET imaging (Fig. 5) demonstrated receptor-specific binding for both compounds. Both Figs. 4 and 5d show not only enhanced tumor accumulation (3.8 *vs*. 1.6 %ID/g, 90 min p.i.) but also high retention of [^68^Ga]FSC(succ-RGD)_3_ in the receptor-positive tumors during the observation period, which is quite similar to the reported [^68^Ga]TRAP(RGD)_3_. In contrast, washout of [^68^Ga]NODAGA-RGD from U87MG xenografts was considerably faster, as can be seen in the dynamic PET images. Whereas for [^68^Ga]FSC(succ-RGD)_3_, no significant loss of image contrast even at 90 min p.i. was found, [^68^Ga]NODAGA-RGD exhibited significant decrease from 35 min p.i. onward.

**Table 2:** Comparison of M21 to tissue ratios of [^68^Ga]FSC(succ-RGD)_3_ with literature data for [^68^Ga]TRAP(RGD)_3_ [13] and [^68^Ga]NODAGA-RGD

	[^68^Ga]FSC(succ-RGD)_3_	[^68^Ga]FSC(succ-RGD)_3_	[^68^Ga]TRAP(RGD)_3_	[^68^Ga]TRAP(RGD)_3_	[^68^Ga]NODAGA-RGD
M21/tissue	1 h	2 h	1 h	2 h	1 h
M21/blood	6.4 ± 2.5	18.7 ± 2.7	19.4 ± 9.1	28.4 ± 19.6	11.3 ± 6.6
M21/spleen	1.1 ± 0.2	1.1 ± 0.1	1.8 ± 0.7	1.3 ± 0.7	0.9 ± 0.4
M21/pancreas	6.0 ± 0.4	10.7 ± 0.8	6.9 ± 1.9	5.7 ± 2.6	6.1 ± 3.1
M21/stomach	1.8 ± 1.3	1.8 ± 0.9	1.4 ± 0.6	1.3 ± 0.8	1.7 ± 0.7
M21/intestine	1.6 ± 0.4	1.9 ± 0.6			1.5 ± 0.7
M21/kidneys	0.5 ± 0.1	0.6 ± 0.1	0.6 ± 0.2	0.5 ± 0.3	1.0 ± 0.4
M21/liver	1.0 ± 0.2	1.4 ± 0.2	1.2 ± 0.5	1.2 ± 0.7	0.8 ± 0.3
M21/heart	3.1 ± 0.8	6.2 ± 0.6	4.7 ± 2.4	3.2 ± 2.7	5.9 ± 2.4
M21/lung	1.5 ± 0.3	2.1 ± 0.1	2.1 ± 0.9	2.2 ± 1.3	
M21/muscle	7.3 ± 0.8	14.4 ± 3.3	6.4 ± 1.9	6.9 ± 3.7	6.1 ± 3.5
M21/M21-L	3.4 ± 0.9	4.3 ± 2.2	4.1 ± 1.4	2.7 ± 1.6	4.0 ± 2.3

The same murine mouse model was used for all compounds. M21 to tissue ratios of [^68^Ga]NODAGA-RGD were calculated based on the literature data [14]

The enhanced tumor accumulation in addition to the good tumor retention for [^68^Ga]FSC(succ-RGD)_3_ not only allows PET imaging at late time points, but also provides the possibility to achieve the same contrast using less radioactivity, which is expected to reduce the radiation burden. Moreover, it is of interest for small animal imaging where the same image quality can be achieved with less radioactivity injected.

Taking all these into account, [^68^Ga]FSC(succ-RGD)_3_ has several advantages compared to [^68^Ga]NODAGA-RGD and possibly to other monomeric RGD peptides. First, the improved tumor accumulation allows for delayed imaging with potentially improved image contrast; second, it provides the potential to use lower activity to reach the same image contrast; third, the high affinity of [^68^Ga]FSC(succ-RGD)_3_ provides the potential for imaging of low-level integrin expression in tissues as Notni *et al*. mentioned [13].

## Conclusion

Three trimeric FSC-RGD conjugates were quantitatively labeled with Ga-68 within 10 min at RT. The Ga-68-labeled compounds showed high stability in challenging solutions and hydrophilic behavior. *In vitro* and *in vivo* studies including PET/CT imaging demonstrated their high affinity for the integrin α_v_β_3_, receptor-selective tumor uptake, rapid predominantly renal excretion, and excellent imaging properties. The comparison of [^68^Ga]FSC(succ-RGD)_3_ and [^68^Ga]NODAGA-RGD shows the advantages of [^68^Ga]FSC(succ-RGD)_3_ in respect of the possibility to carry out delayed imaging with improved tumor to background ratios, imaging with reduced radiation dose, and higher binding affinity allowing monitoring of low-level integrin expression in tissues.

## Electronic supplementary material

Below is the link to the electronic supplementary material.

ESM 1 (PDF 134 kb)
