# Supplementary material for: Comparison of Ga-68-Labeled Fusarinine C-Based Multivalent RGD Conjugates and [68Ga]NODAGA-RGD—In Vivo Imaging Studies in Human Xenograft Tumors
Source: Mol Imaging Biol. 2016 Feb 23;18(5):758–67. doi: 10.1007/s11307-016-0931-3 (PMC5010584; doi:10.1007/s11307-016-0931-3)
Supplement: Supplementary file 1 — (PDF 134 kb) [file 11307_2016_931_MOESM1_ESM.pdf]

Supplemental Information

Comparison of Ga-68 labelled fusarinine C based multivalent RGD conjugates and [^{68}Ga]NODAGA-RGD - in vivo imaging studies in human xenograft tumors

Journal: Molecular Imaging and Biology

Chuangyan Zhai,[1,2] Gerben M. Franssen,[3] Milos Petrik,[4] Peter Laverman,[3] Dominik Summer,[1] Christine Rangger,[1] Roland Haubner,[1] Hubertus Haas,[5] Clemens Decristoforo[1]

[1]Department of Nuclear Medicine, Innsbruck Medical University, Innsbruck, Austria

[2]Department of Nuclear Medicine, Guangdong General Hospital & Guangdong Academy of Medical Sciences, Guangzhou, China

[3]Department of Nuclear Medicine, Radboud University Nijmegen Medical Centre, Nijmegen, The Netherlands

[4]Institute of Molecular and Translational Medicine, Faculty of Medicine and Dentistry, Palacky University, Olomouc, Czech Republic

[5]Division of Molecular Biology, Medical University Innsbruck, Innsbruck, Austria

Corresponding Author: Clemens Decristoforo, PhD, Universitätsklinik für Nuklearmedizin, Medizinische Universität Innsbruck, Anichstr. 35, A-6020 Innsbruck, Austria; Phone: +43 512 504 80951; Fax: +43 512 504 6780951, e-mail: Clemens.Decristoforo@tirol-kliniken.at

Running title: ^{68}Ga-FSC-RGD conjugates for PET imaging

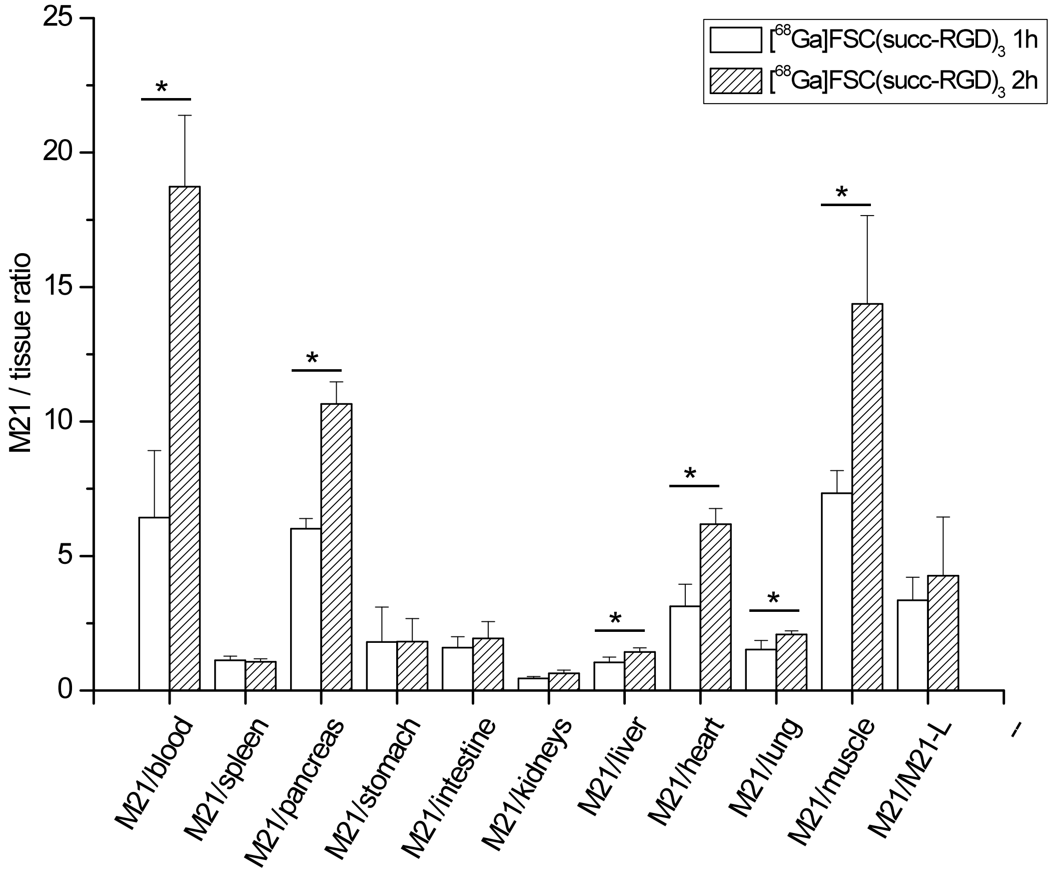

Fig. S1 Comparison of M21-to-tissue ratios of [^{68}Ga]FSC(succ-RGD)$_3$ at 1 and 2 h p.i.. M21/M21-L human melanoma xenografted nude mice were used. Significant differences in uptake are marked with an asterisk (P = 0.05).
